# Hypoxia-Pretreated Human MSCs Attenuate Acute Kidney Injury through Enhanced Angiogenic and Antioxidative Capacities

**DOI:** 10.1155/2014/462472

**Published:** 2014-07-16

**Authors:** Wenbo Zhang, Lirui Liu, Yanhong Huo, Yonghong Yang, Yaping Wang

**Affiliations:** Department of Nephrology, The General Hospital of Beijing Military District, South Mencang 5, Dongcheng District, Beijing 100700, China

## Abstract

Hypoxia preconditioning has been confirmed as an effective strategy to enhance the therapeutic potentials of mesenchymal stem cells (MSCs), such as for myocardial ischemia. However, whether hypoxia preconditioning would produce beneficial effects on MSC-based renal repair has not been demonstrated. In the study, we aimed to determine the feasibility and efficacy of hypoxia preconditioning to enhance MSC-based therapy of acute kidney injury (AKI). MSCs were isolated from human adipose tissues. The paracrine effects of MSCs under normoxia and hypoxia were determined *in vitro*. Rats of AKI were induced by kidney I/R surgery and randomly divided into three groups: I/R control receiving PBS injection; MSC group receiving normal MSC injection; hypoMSC group receiving hypoxia-preconditioned MSC injection. It was demonstrated *in vitro* that paracrine effects of MSCs were significantly enhanced, especially angiogenic factors. Dihydroethidium (DHE) staining showed that antioxidative activities of MSCs were significantly enhanced by hypoxia stimulation. Vascularization, apoptosis, and histological injury were all significantly improved in hypoMSC injected group compared with that in control and MSC injected groups. Finally, the renal function was also significantly improved in hypoMSC injected group compared with that in the other two groups as assessed by the serum creatinine and BUN levels.

## 1. Introduction 

Acute kidney injury (AKI), one of the serious nephropathy, is severely threatening human health [1, 2]. Multifactors may lead to AKI, including arterial occlusion, shock, and organ transplantation. It is reported that AKI is common in critically ill patients, affecting up to 7% of all hospitalized patients [3]. AKI usually lead to great apoptosis/necrosis of renal cells and even renal failure. In those hospital acquired AKI, the mortality rate ranges from 30 to 80% [4]. Though increasing studies have been done during the past decades, there is yet no specific therapy that has significant impact on overall mortality of the disease [5].

Ischemia-reperfusion (I/R) injury is an important cause of AKI [6]. I/R of kidney or renal tissue would result in damage to renal cells via a complex cascade of events, which finally affects the structure and function of kidney [7], such as loss of blood supply, local inflammatory response, and mass production of reactive oxygen species (ROS) [6, 8, 9]. More importantly, kidney could not compensate for these adverse effects by endogenous mechanism [10, 11].

In recent years, transplantation of stem cells has been proposed as a promising strategy for protection of kidney after AKI [4, 10, 12]. Different studies have demonstrated that transplanted stem cells would exert beneficial effects on the ischemic tissues [2, 13, 14]. Among a variety of stem cells, mesenchymal stem cells (MSCs) were investigated the most widely due to their ease of isolation, abundant distribution, low immunogenicity, and so forth [15–17]. Despite various mechanisms for stem cells-based tissue repair (cell fusion, differentiation), secretion of cytokines has been considered as the most important one. It has been demonstrated that cytokines released from MSCs could play multiple roles after ischemia, including scavenging ROS (antioxidative factor), promoting vascularization (bFGF, VEGF), and inhibiting fibrosis (HGH) [13, 18]; all of them could contribute to the repair of ischemic tissues. However, it was also demonstrated that the paracrine effects may be limited under normal condition or have a delay after transplantation in ischemic tissues, during which lots of engrafted cells may die [19], severely impairing their therapeutic potential. Therefore, several strategies have been explored to improve the paracrine effects of MSCs pretransplantation, such as preconditioning and gene modification [20, 21]. Hypoxia preconditioning has been confirmed as a promising one to activate the paracrine capacities of MSCs, especially the angiogenic factors [22, 23]. The strategy has been extensively investigated in therapy of several ischemic diseases and encouraging effects have been achieved [24, 25]. However, whether hypoxia-preconditioning could also exert beneficial effects on MSC-based repair for AKI is still not documented.

In present study, we aimed to determine whether hypoxia preconditioning could exert beneficial effects on MSC-based therapy of AKI. Rats of AKI were induced by kidney I/R surgery. Then, MSC or hypoxia-preconditioned MSC was injected into the left kidney cortex; PBS was used as control. The production of ROS in I/R renal tissues was determined by dihydroethidium (DHE) staining. Vascularization, apoptosis, tissue damage, and function of ischemic kidneys were evaluated by multimodality methods.

## 2. Materials and Methods

### 2.1. Animals and Experimental Design

Male adult SD rats were housed in cages and were fed a standard laboratory chow and water ad libitum. All procedures were consistent with the guidelines and policies of the Hospital Animal Care and Use Committee. Each procedure was optimized to minimize the pain of animals.

A total of 75 rats were used in the study (*n* = 25 for each group). Animals were randomly divided into four groups: animals receiving 100 *μ*L saline injection after AKI; animals receiving 2 × 10^6^ human adipose-derived MSCs (in 100 *μ*mL saline) injection after AKI; animals receiving 2 × 10^6^ hypoxia-preconditioned human adipose-derived MSCs (in 100 *μ*mL saline) after AKI. During the experiment, two rats died before the end. Two other rats were used so that each group still has 25 animals.

### 2.2. Cell Isolation, Cultivation, and Characterization

Adipose tissues were obtained from raw human lipoaspirates. Human adipose-derived MSCs were isolated as the previous report [16, 26]. Briefly, lipoaspirates were washed with PBS to remove contaminating debris and red blood cells. Then, the tissues were digested with 0.1% collagenase I (Sigma) in serum-free *α*MEM for 45 min. After that, the digestion was terminated by adding equal volume of *α*MEM/10% fetal bovine serum (FBS, Gibco). The liquid was filtered by 80 *μ*m mesh and then centrifuged at 800 g for 8 min. The cell pellet was resuspended in *α*MEM supplemented with 10% FBS. The cells were seeded onto tissue culture plates and cultured in 37°C, 5% CO_2_ incubator.

The immunophenotype of MSCs was determined by flow cytometry according to previous reports [2]; CD29, CD90, CD45, CD34, and CD31 were analyzed. The multipotency was confirmed by osteogenic and adipogenic differentiation according to the previous report [16].

### 2.3. Determination of the Paracrine Factors

10^6^ MSCs were seeded on culture plates and cultured in normoxic (21% O_2_) or hypoxic (1% O_2_) conditions for 24 h. Then, the medium was collected for growth factor assay; bFGF, VEGF, and HGF were determined by ELISA array. Data was expressed as mean ± SEM pictograms (pg) of the factor per 10^6^ MSCs.

### 2.4. Cell Viability Analysis

Cell viability after hypoxic treatment was detected by flow cytometry. Briefly, cells were collected and incubated with propidium iodide (PI) solution. After that, cells were analyzed by flow cytometry.

### 2.5. The Induction of AKI and Cell Transplantation

The acute renal ischemia/reperfusion (I/R) injury was induced by transiently clamping bilateral renal pedicles as previously reported [2]. Briefly, rats were anesthetized with intraperitoneal injection of sodium pentobarbital (30 mg/kg). Laparotomy was performed and kidneys were exposed. Bilateral renal pedicles were clamped with atraumatic vascular clamps for 40 min followed by reperfusion. To prepare hypoxia-treated MSCs (hypoMSCs), MSCs were cultured under normal condition. When they reached 80% confluence, cells were then transferred into hypoxic conditions (1% O_2_) and incubated for 24 h. After that, cells were collected for the following transplantation surgery.

At the time of reperfusion, 100 *μ*L saline or 2 × 10^6^ MSCs or 2 × 10^6^ hypoxia-preconditioned MSCs (*n* = 25/group) were injected into the left kidney cortex using a 28-gauge needle. The abdomen was then closed and rats were allowed to recover with cautious care.

### 2.6. Dihydroethidium Staining

Animals were sacrificed at day 1 after surgery (*n* = 5 for each group). Kidneys were rapidly excised and frozen in optimal cutting temperature freezing medium for preparation of cryosections. Dihydroethidium (DHE) staining was performed on the cryosections to determine the ROS levels according to the previous report [2]. Poststaining sections were examined under fluorescence microscopes (Olympus). The intensity of DHE staining was quantified according to the previous report [2].

### 2.7. Histological Analysis

The kidney samples were collected at 3 days and 1 week (*n* = 5 for each group) after surgery and fixed in 4% paraformaldehyde. 4 *μ*m paraffin-embedded sections were prepared with standard protocols. Hematoxylin and eosin (H&E) staining was performed for tubular injury analysis. The examination and scores of the sections were carried out by an experienced technician who was blind to the experiment [12]. The tubular injury was graded based on the degree of tubular necrosis, loss of brush border, cast formation, and tubular dilatation, as in previous reports [2, 6]. The score standard was as follows: 0, normal kidney; 1, minimal injury (<5% involvement); 2, mild injury (5~25% involvement); 3, moderate injury (25~75% involvement); and 4, severe injury (>75% involvement).

### 2.8. Immunohistochemistry

For analysis of vascular density, immunohistochemical staining was performed on paraffin-embedded kidney sections using anti-vWF antibodies (Sigma) according to previous reports [2]. Then, the vascular structures were counted under microscope; 5 random fields on each section were counted. The vascular density was expressed as vessels/field.

### 2.9. TUNEL Staining

At one week, the apoptosis of renal cells was assessed based on terminal deoxynucleotidyl transferase dUTP nick-end labeling (TUNEL) assay. As described above, 4 *μ*m paraffin-embedded sections were prepared for assay. TUNEL staining was performed according to manufacturer's guideline. Then, TUNEL-stained sections were observed under light microscopy; the positive-stained cells were counted and compared among different groups.

### 2.10. RT-PCR

Total RNA was extracted using RNAprep pure Cell/Bacteria Kit (TIANGEN) according to manufacturer's instruction. Reverse transcription was performed using standard procedures to synthesize first-strand cDNA. The gene-specific primers were designed using primer3.0 and used in the PCR amplification. PCR product was detected by agarose electrophoresis.

### 2.11. Western Blotting

Kidney samples were obtained and homogenized with a rotor stator. Then, tissues were lysed in Laemmli sample buffer (Bio-Rad). Proteins were collected and concentrations were determined using the BCA Protein Assay Kit (Thermo Scientific). Proteins were loaded on a 12–15% sodium dodecyl sulfate polyacrylamide gel for electrophoresis. Then, proteins were transferred to a PVDF membrane (Roche). After blocking with 5% nonfat dried milk (in TBST), the primary antibody was added and incubated overnight at 4°C. Unconjugated antibodies were removed by washing with TBST; the membrane was further incubated with horseradish peroxidase-conjugated secondary antibodies for 1 h at room temperature (Cell Signaling Technology). The protein bands were detected by enhanced chemiluminescence reagent (Applygen).

### 2.12. Renal Function Assay

To assess the renal function, blood was taken at 1 d, 3 d, 14 d, and 28 d after the surgery for each rat (*n* = 10 for each group). Serum levels of creatinine and blood urea nitrogen (BUN) were measured and compared between the four groups. Measurement of creatinine and BUN level was performed with standard protocol in our hospital.

### 2.13. Statistical Analysis

All data are expressed as mean ± SD. Statistical analyses were performed with SPSS 17.0 software. Comparison between four groups was performed using Student's *t*-test. A value of *P* < 0.05 was considered statistically significant.

## 3. Results 

### 3.1. Characteristics of Human Adipose-Derived MSCs

**Figure 1:**
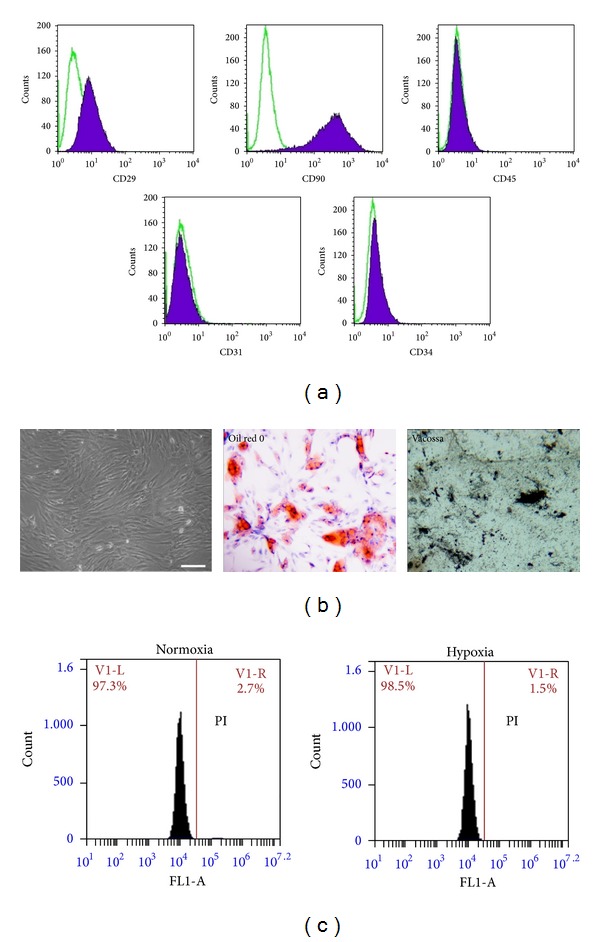
Isolation, cultivation, and characterization of human adipose-derived MSCs. (a) Flow cytometry analysis of immunophenotypes of human adipose-derived MSCs. Most MSCs expressed CD29 and CD90 and do not express CD45, CD31, and CD34; (b) morphology and multidifferentiation of MSCs. Human adipose-derived MSCs demonstrated fibroblast-like morphology in culture, and they could differentiate into adipogenic and osteogenic lineages when cultured in differentiation medium. Adipogenic differentiation was confirmed by oil red O staining and osteogenic differentiation was confirmed by von Kossa staining (Bar = 100 *μ*m). (c) Flow cytometry analysis of cell viability after PI staining.

After three passages, flow cytometry analysis demonstrated that most MSCs were positive for CD29 and CD90 and negative for CD31, CD34, and CD45 (Figure 1(a)). This is consistent with previous report [16]. In normal culture (37°C, 5% CO_2_), human adipose MSCs exhibited fibroblast-like morphology (Figure 1(b)). When cultured in adipogenic and osteogenic differentiation medium, MSCs differentiated into adipose and osteoplastic cells that were positive to oil red O staining and von Kossa staining, respectively.

**Figure 2:**
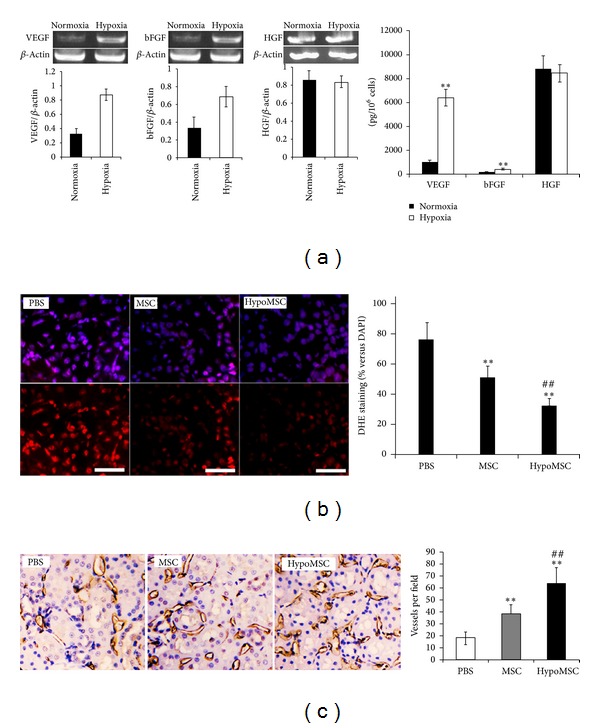
Effects of hypoxia preconditioning on the angiogenic and antioxidative capacities of MSCs* in vitro* and in kidney. (a) RT-PCR demonstrated that the transcription of angiogenic genes in MSCs was increased when cultured in hypoxia, while Elisa assay obtained consistent results from protein level; (b) DHE staining showed that hypoMSCs exhibited higher antioxidative activity than control MSCs after transplantation in I/R kidney; (c) angiogenic effects in I/R kidney by hypoMSCs. **P < 0.01 compared with normaxia or PBS group; ^##^ *P* < 0.01 compared with control MSCs. HypoMSC indicates hypoxia-preconditioned MSCs. Bar = 50 *μ*m.

After hypoxic treatment, cell viability analysis by flow cytometry showed that MSC viability was maintained at a high level. No significant cell death was found between hypoxia-treated MSCs and normal MSCs (Figure 1(c)). The paracrine effects of MSCs were compared under normaxia and hypoxia condition. RT-PCR demonstrated that transcription of angiogenic factors, bFGF and VEGF, was significantly higher in hypoxia-preconditioned MSCs (hypoMSC) than control MSCs (cultured under normaxia condition), but HGF, an antifibrotic factor, was not influenced by hypoxia. Elisa assay obtained consistent results with RT-PCR that the secreted bFGF and VEGF from MSCs under hypoxia were significantly higher than that under normoxia, but antifibrotic factor HGF (Figure 2(a)). The results demonstrated that hypoxia preconditioning selectively unregulated the expression of angiogenic factors. In addition, we also analyzed the hypoxia marker HIF-1*α* (hypoxia inducible factor-1*α*) expression by RT-PCR, but the factor was not upregulated in mRNA level (Data not shown). As is known, oxygen regulation of HIF-1*α* is essentially produced at protein level. Prolyl hydroxylases (PHD) modify HIF-1*α* proteins for their rapid degradation. Hypoxia leads to inhibition of PHDs and allows for the presence of HIF-1*α* proteins. Therefore, we further analyzed the HIF-1*α* expression by western blotting and found that it was increased in protein level (data not shown).

### 3.2. Influences of MSCs on the Oxidative Level and Vascularization after Renal I/R Injury

The increase in superoxide production after I/R is associated with augmented oxidative stress, which is a main cause of I/R injury. It has been demonstrated that factors released from stem cells could significantly scavenge superoxides in ischemic tissues [14]. Therefore, the superoxide levels were determined 24 h after MSC transplantation. According to previous reports [26], dihydroethidium (DHE) is a reagent to measure superoxide levels. In the study, we repeated the DHE staining to determine the superoxide levels after renal I/R injury. The results demonstrated that the intensities of DHE staining in kidney receiving MSC transplantation were significantly lower than that in control (*P* < 0.01, Figure 2(b)). Importantly, hypoMSCs were more effective than control MSCs in superoxide scavenging, suggesting that antioxidative capacity of MSCs was enhanced due to improved activated paracrine effects by hypoxia.

To determine the effects of hypoMSCs on angiogenesis in ischemic kidney tissues, paraffin-embodied kidney sections were prepared 4 w after MSC transplantation. As shown in Figure 2(c), transplantation of MSCs significantly improved vascular densities in ischemic kidney tissues compared with saline, while the vascular density in kidney receiving hypoMSCs injection was higher than that receiving MSC injection, indicating that activation by hypoxia precondition significantly enhanced angiogenic effects of MSCs in ischemic kidney tissues.

### 3.3. Apoptosis of Renal Cells

**Figure 3:**
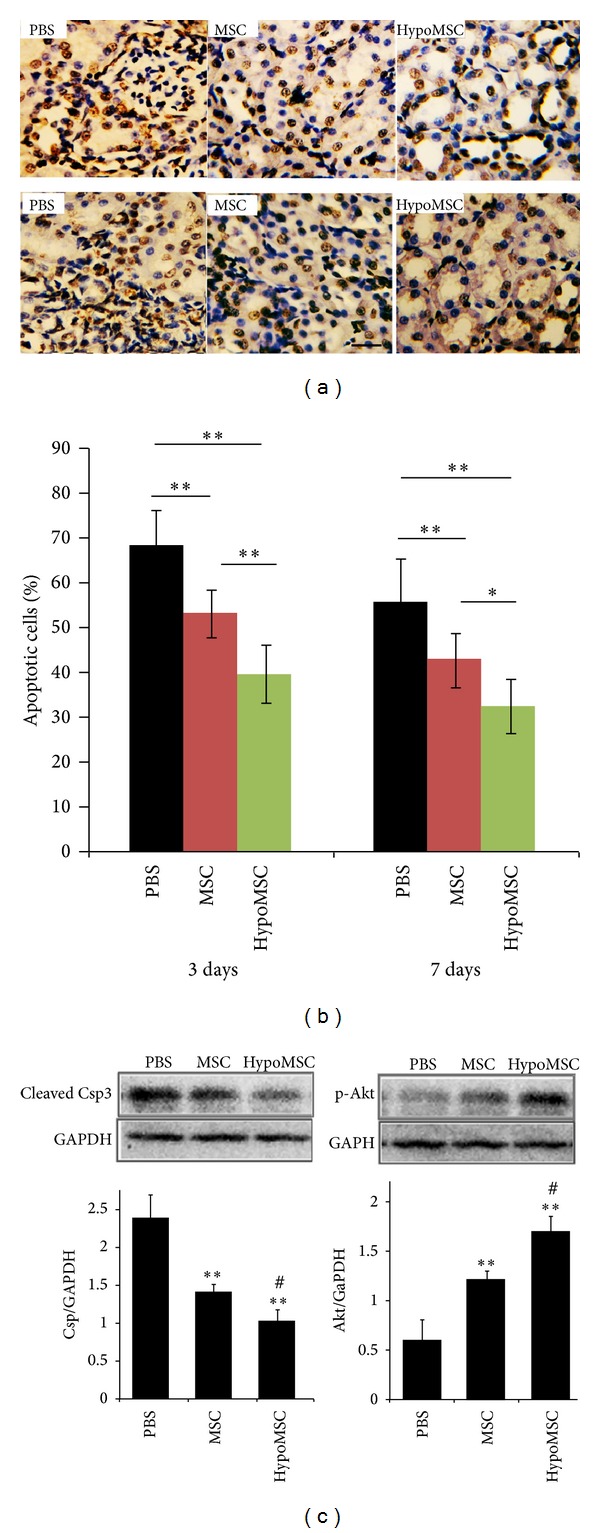
Antiapoptotic and histopathological analysis of kidney injury after I/R. (a) TUNEL staining 3 days and one week after surgery; (b) quantification of apoptotic cells in renal tissues; (c) western blotting analysis of cleaved caspase 3 and p-Akt in renal tissues one week after surgery.

After I/R and MSC injection, we performed TUNEL staining to determine apoptosis in I/R kidney. After TUNEL staining, kidney sections were observed under light microscopy and positively stained cells were quantified in each group. As shown in Figures 3(a) and 3(b), apoptotic cells were abundantly distributed in control group, while in MSC injection group, the apoptotic cells were significantly decreased compared with that in control (*P* < 0.01). Furthermore, compared with MSC injection group, apoptotic cells were further decreased in hypoMSCs injection group (*P* < 0.01).

Western blotting analysis was performed to provide additional evidence for cell apoptosis. Detection of apoptotic protein caspase 3 (Csp3) and prosurvival protein Akt was performed at 1 week and we obtained consistent results with TUNEL staining. As shown in Figure 3(c), I/R surgery induced high level expression of cleaved caspase 3, which was accompanied with low level phosphorylation of Akt. The results suggested that I/R induced significant apoptosis to renal cells. Compared with I/R control, the expression of cleaved csp3 was significantly lowered in MSC injection group (*P* < 0.01) while the phosphorylation of Akt was significantly raised (*P* < 0.01), indicating that injection of MSC significantly protected renal cells against apoptosis. Then, the antiapoptotic effects were further compared between MSC injection group and hypoMSCs injection groups. The results showed that injection of hypoMSCs further lowered cleaved csp3 expression while it improved Akt phosphorylation compared with normal MSCs (*P* < 0.01).

### 3.4. Histology of I/R Kidney Tissue

**Figure 4:**
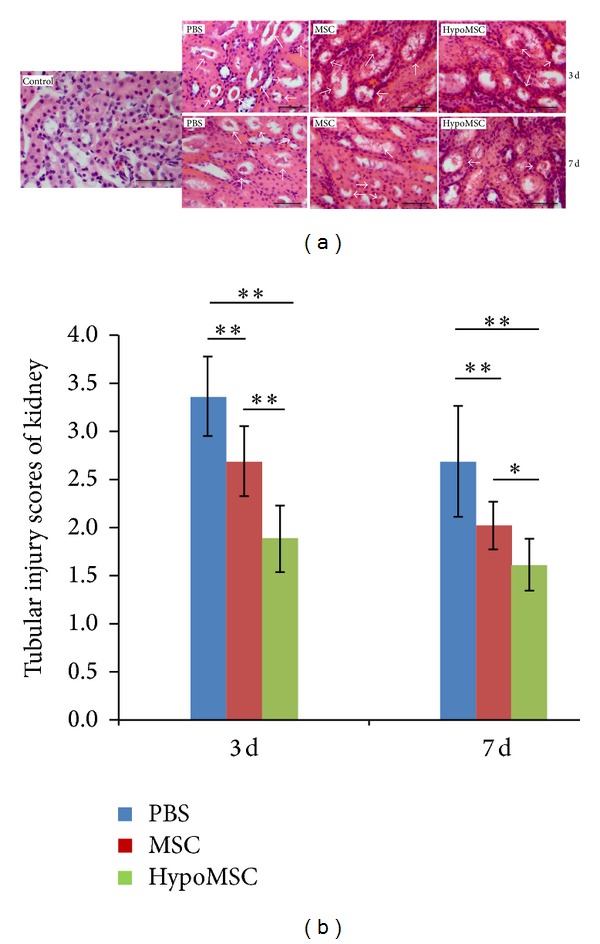
H&E staining of kidney tissues. (a) The microscope observation of tissue injury at 3 days and 1 w after surgery (Bar = 50 *μ*m). (b) Scores of renal injury in different groups after surgery. *P < 0.05; **P < 0.01.

Three days and one week after renal I/R surgery, kidney tissue specimens from each group were obtained. The tissue sections were stained by H&E staining and then assessed. Under light microscopy, significant tubular cell necrosis, cytoplasmic vacuoles, loss of brush border, and tubular dilatation could be observed in PBS injection group (Figure 4(a)), indicating that marked renal injury was induced by I/R. Compared with PBS control, injection of MSCs after surgery significantly attenuated I/R induced renal injury. Under microscopy, less tubular cell necrosis and loss of brush border were observed, tubular dilatation was of lower grade than that of I/R control too. Quantitative analysis showed that histological scores for renal injury in MSC injection (including normal MSCs and hypoMSCs) groups were significantly lower than that in I/R control group (both *P* < 0.01, Figure 4(b)). Likewise, we further compared the histology scores between normal MSCs injection and hypoMSCs injection. Further improvement of histological morphology could be observed in hypoMSCs group compared with that in MSCs group. The histological scores of renal injury in hypoMSCs group were also significantly lower than MSCs group (*P* < 0.01).

### 3.5. Renal Function

**Figure 5:**
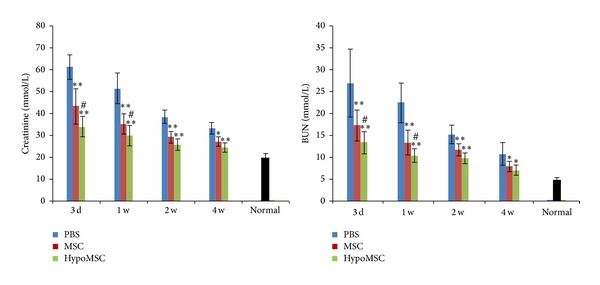
Renal function after I/R. Blood level of creatinine and BUN measured at different time after kidney I/R for evaluation of renal function. **P < 0.01 compared with PBS control; *P < 0.05 compared with PBS control; ^#^ *P* < 0.05 compared with MSC group.

At 3 days, 1 w, 2 w, and 4 w after surgery, the effects of MSC injection on renal function were evaluated. The serum levels of creatinine and BUN in each group were measured. As shown in Figure 5, a high level of creatinine and BUN was detected in PBS group, indicating that I/R surgery has induced the renal I/R injury. In comparison, both creatinine and BUN levels were significantly lowered in MSC injection group at each time point (including MSC and hypoMSC injection). Injection of hypoMSCs especially was more effective than MSCs.

## 4. Discussion

Acute kidney I/R is associated with significant renal injury, which may be partially attributed to the lack of blood supply, mass production of superoxides, and so forth [27, 28]. Different groups have demonstrated that transplantation of MSCs could exert beneficial effects on injured kidney tissues [10, 12]. However, delayed paracrine effects of transplanted MSCs were one of main limitations for their therapeutic potential [19]. Hypoxia preconditioning was confirmed by several groups as an effective strategy to activate MSCs and enhance their therapeutic efficacy for ischemic disease [20]. In the study, we firstly investigated whether hypoxia preconditioning could enhance MSC-based therapy of AKI. We demonstrated that hypoxia preconditioning significantly enhanced secretion of angiogenic factors from MSCs, which significantly contributed to the superoxide scavenging, angiogenesis, and antiapoptosis of MSCs in injured kidney. The study confirmed the feasibility and efficacy of hypoxia preconditioning to enhance MSC-based therapy for AKI.

In previous reports, several strategies have been investigated to enhance the therapeutic potential of MSCs, including (1) gene modification, that is, making MSCs overexpress cytokine genes or antiapoptotic genes, which significantly improved their therapeutic capacities or survival in ischemic tissues [21, 29]; (2) cotransplantation with adjuvant, such as adjuvant drugs or biomaterials, which may significantly enhance the retention or survival of transplanted cells [2, 26]; (3) preconditioning before transplantation with drugs, cytokines, or culture condition [30]. Gene modification is accompanied with risks to activate cancer genes and thus should be very cautious about* in vivo* application; cotransplantation with adjuvants is promising in future application; it could improve the ischemic microenvironment assisting the retention and survival of stem cells, but it fails to stimulate the therapeutic potentials of stem cells. Preconditioning before transplantation is effective to stimulate the therapeutic potentials of stem cells. Combined application of adjuvants and preconditioning would exert more beneficial effects on stem cell therapy and deserved in-depth investigation.

This study demonstrated that angiogenic factors, such VEGF and bFGF, were significantly enhanced in MSCs by hypoxia treatment. This may explain the* in vivo* finding that revascularization in hypoMSC-treated kidneys was the most significant (Figure 2(c)). We also found that the antioxidative capacity of MSCs was also improved by hypoxia preconditioning; this is novel in the study. We supposed that this may be attributed to the increased secretion of antioxidative cytokines by hypoxia stimulation. However, we failed to reveal these factors in the study. In terms of antiapoptotic capacity, the study showed that hypoMSCs further inhibited apoptosis of renal cells compared with normal MSCs as detected by TUNEL staining. To further confirm the antiapoptotic effects of hypoMSCs, we detected the expression of apoptosis-related proteins. Akt is a prosurvival factor for cells. Activated Akt (p-Akt) plays an important role in antiapoptosis; caspase 3 is a proapoptotic protein; a lot of cell death was mediated by cleaved caspase 3 (activated caspase 3). In the study, we demonstrated that transplantation of hypoMSCs significantly increased the level of p-Akt, while it decreased the level of cleaved caspase 3 compared with the other groups, indicating that hypoMSCs may upregulate the prosurvival signals and downregulate apoptotic signals in ischemic kidney. This is consistent with the results from TUNEL staining. Our explanation for this included the following: (1) hypoxia preconditioning may also activate the antiapoptotic capacity of MSCs, resulting in more antiapoptotic cytokine release; (2) the enhancement of antioxidative and angiogenic capacities of transplanted MSCs contributed to the survival of renal cells. Collectively, antioxidative, antiapoptotic, and angiogenic capacities, all of them, contributed to the I/R kidney protection of hypoMSCs and thus better histological (H&E staining) and functional consequences (biochemical data, serum creatinine, and BUN) were observed after hypoMSC transplantation.

Different mechanisms may exist underlying the observation that transplanted MSCs could modify all kidney effects of I/R, such as cell differentiation and paracrine effects. However, it has been demonstrated by independent groups that rare MSCs would differentiate into target cells* in vivo*, and significant therapeutic effects could also be obtained with MSC-conditioned medium without MSCs [29, 31–33]. Therefore, we supposed that the paracrine effects should be the main mechanism through which MSCs modify all kidney effects of I/R as observed in the present study.

In conclusion, the study revealed that hypoxia preconditioning significantly enhanced the therapeutic effects of human adipose-derived MSCs on AKI. Further, we confirmed that these enhanced therapeutic potentials by hypoxia preconditioning were, at least partially, attributed to the enhanced antioxidative, antiapoptotic, and angiogenic capacities of MSCs by hypoxia stimulation. The study confirmed the feasibility and efficacy of hypoxia preconditioning in MSC-based therapy for AKI and provided promising strategy in application of MSCs after renal injury.
